# Serum PlGF and EGF are independent prognostic markers in non-metastatic colorectal cancer

**DOI:** 10.1038/s41598-019-47429-5

**Published:** 2019-07-29

**Authors:** Sebastian Schölch, Andreas Bogner, Ulrich Bork, Mohammad Rahbari, Balázs Győrffy, Martin Schneider, Christoph Reissfelder, Jürgen Weitz, Nuh N. Rahbari

**Affiliations:** 1 Department of Surgery, Universitätsmedizin Mannheim, Medical Faculty Mannheim, Heidelberg University, Mannheim, Germany; 2 Department of Gastrointestinal, Thoracic and Vascular Surgery, Medizinische Fakultät Carl Gustav Carus, Technische Universität Dresden, Dresden, Germany; 3 German Cancer Consortium, Heidelberg, Germany; 4 German Cancer Research Center (DKFZ), Heidelberg, Germany; 5 MTA TTK Lendület Cancer Biomarker Research Group, Magyar Tudósok körútja 2., H-1117 Budapest, Hungary; 6 Semmelweis University, 2nd Department of Pediatrics, Bókay u. 53-54., H-1083 Budapest, Hungary; 7 Department of General, Gastrointestinal and Transplant Surgery, University Hospital Heidelberg, Medical Faculty Heidelberg, Heidelberg University, Heidelberg, Germany

**Keywords:** Colorectal cancer, Tumour biomarkers, Surgical oncology

## Abstract

The aim of this study was to determine the prognostic value of circulating angiogenic cytokines in non-metastatic colorectal cancer (CRC) patients. Preoperative serum samples of a training (TC) (n = 219) and a validation cohort (VC) (n = 168) were analyzed via ELISA to determine PlGF, EGF, VEGF, Ang1, PDGF-A, PDGF-B, IL-8 and bFGF levels. In addition, survival was correlated with PlGF and EGF expression measured by microarray and RNAseq in two publicly available, independent cohorts (n = 550 and n = 463, respectively). Prognostic values for overall (OS) and disease-free survival (DFS) were determined using uni- and multivariate Cox proportional hazard analyses. Elevated PlGF is predictive for impaired OS (TC: HR 1.056; *p* = 0.046; VC: HR 1.093; *p* = 0.001) and DFS (TC: HR 1.052; *p* = 0.029; VC: HR 1.091; *p* = 0.009). Conversely, elevated EGF is associated with favorable DFS (TC: HR 0.998; *p* = 0.045; VC: HR 0.998; *p* = 0.018) but not OS (TC: *p* = 0.201; VC: *p* = 0.453). None of the other angiogenic cytokines correlated with prognosis. The prognostic value of PlGF (OS + DFS) and EGF (DFS) was confirmed in both independent retrospective cohorts. Serum PlGF and EGF may serve as prognostic markers in non-metastatic CRC.

## Introduction

With an estimated 1.4 million new cases and 700.000 deaths worldwide, colorectal cancer (CRC) is among the three most frequent malignant diseases^1^. Although the worldwide incidence is declining due to better early detection and treatment methods, CRC remains one of the leading causes of cancer-related death^2^, mainly due to synchronous or metachronous distant metastases^3^. While many patients never experience recurrence after curative resection, others suffer from recurrence and ultimately succumb to the disease. It therefore remains an important task to develop new prognostic markers and techniques in order to identify high risk patients requiring aggressive treatment and avoid overtreatment in low risk disease^4–6^.

Neoangiogenesis is essential for tumor growth and metastatic spread. Therefore, angiogenic factors are important targets of anti-tumor therapy. There is a broad panel of known cytokines involved in tumor angiogenesis including vascular endothelial growth factor (VEGF), Angiopoietin 1 (Ang1), platelet-derived growth Factor A and B (PDGF-A, PDGF-B), the chemokine Interleukin 8 (IL-8) and basic fibroblast growth factor (bFGF)^7–9^.

Placental growth factor (PlGF) is a member of the vascular endothelial growth factor (VEGF) family and a key molecule in angiogenesis and vasculogenesis^10,11^; however, its molecular mechanisms of action remain incompletely understood^12^. Aside from its role in pregnancy, preeclampsia and early pregnancy loss detection, PlGF may also be involved in pathologic and physiological responses to hypoxia^13^. Elevated PlGF levels are found in several inflammatory diseases and are involved in the recruitment and homing of regulatory immune cells^14,15^. PlGF thus contributes to immune escape mechanisms by suppressing anti-tumor immunity within solid malignancies^16^. Another tumor-promoting mechanism of PlGF is its binding capacity to vascular endothelial growth factor receptor 1 (VEGFR1), which is a known tumor driver and upregulated in a variety of different malignant tumors^17^. Its activation triggers angiogenesis, tumor spread and growth. As a result, VEGF(R) inhibitors are widely used in the treatment of many solid tumors including metastatic CRC^18^.

Epidermal growth factor receptor (EGFR) is a member of the HER/ERBB family of receptor tyrosine kinases and responsible for downstream activation of several oncogenic mechanisms including angiogenesis, endothelial cell invasion, proliferation and migration. Epidermal growth factor (EGF) is the main activator of EGFR and its downstream signaling cascade^19,20^.

There are only few studies that tested serum levels of PlGF and EGF in patients with CRC indicating PlGF and EGF as potential prognostic markers of tumor recurrence and survival^21,22^. However, the currently available data on these two factors was derived mainly from stage IV patients. In addition, serum PlGF and EGF were primarily evaluated for their predictive rather than their prognostic value. The aim of this study was therefore to evaluate the serum levels of a panel of angiogenic factors including PlGF and EGF as potential prognostic markers in patients with non-metastatic CRC.

## Methods

### Patients

This report is in accordance with the REMARK guidelines^23^. Informed consent to this study was obtained from every patient prior to enrollment. A total of 387 CRC patients were recruited between 08/1997 and 05/2010 and divided into a training cohort (TC; n = 219; 1997–2007) and a validation cohort (VC; n = 168; 2007–2010). Patients were prospectively enrolled in this study and underwent surgery with curative intent at the Department of Surgery, University of Heidelberg, Germany for histologically proven, non-metastatic CRC. Preoperative staging included colonoscopy with biopsy and a CT scan of the chest and abdomen to exclude metastatic disease. Surgical procedures included (extended) right and left hemicolectomy, transverse colon resection, sigmoid colon resection, anterior and abdominoperineal rectal resection. All adjuvant and neoadjuvant treatments were given according to German S3 guidelines for colorectal cancer and did not change during the patient recruitment period. Neoadjuvant therapy was only given to rectal cancer patients (mid or lower rectum) as short-course chemoradiation (5 × 5 Gy + 5-Fluorouracil) without additional antibodies, particularly no anti-EGFR antibodies.

Adjuvant therapy was given only to patients with UICC III or high risk UICC II (T4 tumors, L1, V1, emergency surgery for tumor-related bowel obstruction). Adjuvant therapy consisted of 6 months of 5-FU +/− oxaliplatin/leucovorin without additional antibodies.

The study was approved by the institutional ethics board of the Faculty of Medicine of the University of Heidelberg and was conducted in accordance with the Declaration of Helsinki as well as the ICH Harmonized Tripartite Guideline for Good Clinical Practice in their most current versions.

### Sample collection

Serum samples from all patients were drawn in the operating room prior to the first incision from a central venous catheter. Standard 9 mL serum collection tubes (S-Monovette; Sarstedt, Nümbrecht, Germany) were used for sample collection, immediately transferred to the laboratory, centrifuged, aliquoted and stored at −80 °C.

### ELISA

Enzyme-linked immunosorbent assays (ELISA) were used to determine the serum levels of human PlGF, human EGF, human VEGF, human Ang1, human PDGF-A, human PDGF-B, human IL-8 and human bFGF using commercially available kits (Quantikine, R&D Systems, Minneapolis, MN, USA) according to the manufacturer’s instructions. Data collection was carried out on an Infinite 200 Pro ELISA reader (Tecan, Männedorf, Switzerland).

### Affymetrix CRC cohort

A database of publicly available CRC patient samples measured by Affymetrix gene chips was set up as described previously^24^. In brief, gene chip datasets with transcriptome-wide gene expression data generated by Affymetrix gene arrays and available survival data were identified in the Gene Expression Omnibus repository (www.ncbi.nlm.nih.gov/geo/). Samples were MAS5 normalized and a second scaling normalization was performed to set the mean expression across all probes to 1000. We selected the probe sets 209652_s_at for PlGF and 206254_at for EGF.

### TCGA CRC cohort

CRC patients measured by RNA-seq were published in The Cancer Genome Atlas (TCGA)^25^. Pre-processed level 3 data generated using Illumina HiSeq. 2000 RNA Sequencing V2 and probe IDs 5228 (PlGF) and 1950 (EGF) were used. For each sample, the expression level was determined using a combination of MapSplice and RSEM. The individual sample files were merged in R using the plyr package^26^.

### Statistical analysis

All values are reported with standard deviation (SD). For univariate analyses, categorical variables were compared using the *χ*^2^*-test*. Continuous variables were expressed as arithmetic mean with standard deviation and compared using students’ t-test. All variables with *p* < 0.05 were included in a stepwise backward, multivariate logistic regression model using the median as a cutoff. Survival analysis was performed by employing Cox proportional hazard regression in the R statistical environment (www.r-project.org) as described previously^27^. Hazard ratios with 95% confidence intervals and log-rank P values were calculated using the library “survival”. Overall survival, defined as time to death, and disease-free survival, defined as time to recurrence, were determined. Kaplan-Meier curves were drawn to visualize the survival differences. All variables with p < 0.05 on univariate analysis were added to a multivariate Cox regression model adjusting for age, sex, site of disease (SOD) and UICC stage and addressed for multiple testing controlling the false discovery rate by using the Benjamini-Hochberg procedure^28^. Statistical analyses were carried out using IBM SPSS Statistics v23 (SPSS Inc., Chicago, IL), R (www.r-project.org) and GraphPad Prism v7 (Graph Pad Software Inc., La Jolla, CA).

### Patient demographics

Characteristics of the study cohorts are listed in Table 1. There were 127 male (58.0%) and 92 female (42.0%) patients with a mean age of 67.0+/− 11.1 years in the TC. The VC included 102 male (60.7%) and 66 (39.3%) female patients aged 67.0+/− 10.8 years. All patients had histologically confirmed postoperative negative resection margins (R0). There were no significant differences in any demographic, clinical or histological variables between patients in the TC and VC.

**Table 1:** Patient characteristics of the training and validation cohorts.

	Training cohort (n = 219)	Validation cohort (n = 168)	*p*
	*n* (*%*); *mean* (*SD*)	*n* (*%*); *mean* (*SD*)	
**Sex**			
Male	127 (58.0)	102 (70.7)	0.598
Female	92 (42.0)	66 (39.3)	
Age [years]	67.0; 11.1	67.0; 10.8	0.650
≤70	137 (62.6)	107 (63.7)	0.819
>70	82 (37.4)	61 (36.3)	
**Body mass index [kg/cm** ^**2**^ **]**			
≤25	77	76	0.612
>25	104	92	
**Site of disease**			
Colon	106 (48.4)	72 (42.9)	0.278
Rectum	113 (51.6)	96 (57.1)	
**Surgical procedure**			
(Ext.) right hemicolectomy	49 (22.4)	41 (24.4)	
Transvere colon resection	1 (0.5)	1 (0.6)	
(Ext.) left hemicolectomy	24 (11.0)	15 (8.9)	
Sigmoid resection	13 (5.9)	15 (8.9)	
Anterior rectal resection	109 (49.7)	83 (49.4)	
Rectal extirpation	9 (4.1)	11 6.5)	
Others	14 (6.4)	2 (1.2)	
**Neoadjuvant therapy**			
Yes	47 (21.5)	43 (25.6)	0.340
No	172 (78.5)	125 (74.4)	
**pT**			
pT0/pTis	1 (0.5)	3 (1.8)	0.198
pT1	22 (10.0)	14 (8.3)	
pT2	68 (31.1)	42 (25.0)	
pT3	113 (51.6)	101 (60.1)	
pT4	15 (6.8)	8 (4.8)	
**pN**			
N0	134 (61.2)	109 (64.9)	0.411
N1	54 (24.7)	40 (23.8)	
N2	31 (14.2)	18 (10.7)	
Nx	0	1 (0.6)	
**cM**			
0	219 (100)	168 (100)	—
1	0	0	
**UICC stage**			
I	69 (31.5)	45 (26.8)	0.386
II	65 (29.7)	65 (38.7)	
III	85 (38.8)	58 (34.5)	
**Grade**			
1	0	0	0.498
2	5 (2.3)	2 (1.2)	
3	155 (70.8)	115 (68.5)	
4	44 (20.1)	25 (14.9)	
x	15 (6.8)	26 (15.4)	

Abbreviations: SD, standard deviation; BMI, body mass index; Ext., extended; UICC, Union Internationale Contre le Cancer; PlGF, Phosphatidylinositol-glycan biosynthesis class F protein/Placental growth factor; EGF, epidermal growth factor.

## Results

### Association of angiogenic factors with clinicopathologic variables

The arithmetic mean of serum PlGF levels was 15.5 pg/mL (+/− 5.8) in the TC and 14.4 pg/mL (+/− 4.5) in the VC (p = 0.042). Serum EGF levels in the TC were 251.8 pg/mL (+/− 175.2) and 343.3 pg/mL (+/− 197.2) in the VC (*p* < 0.001). Neither in the TC (*r* = − 0.180, *p* = 0.023) nor in the VC (*r* = − 0.027, *p* = 0.728) a strong correlation between EGF and PlGF serum levels was observed. Associations of serum PlGF and EGF with clinicopathologic variables on univariate analysis are shown in Table 2. Univariate analysis and arithmetic means for VEGF, Ang1, PDGF-A, PDGF-B, IL-8 and bFGF are given in Supplementary Tables 1–3. The multivariate logistic regression models were adjusted for age, sex, SOD and UICC stage (Table 3). Higher serum PlGF levels were observed in patients with age > 70 in VC (Odds Ratio (OR) 0.425; 95% Confidence Interval (CI): 0.215–0.840; *p* = 0.014), BMI > 25 in VC (OR 0.439; 95% CI 0.226–0.852; *p* = 0.015) and neoadjuvant chemotherapy (TC: OR 0.457; 95% CI 0.222–0.940; *p* = 0.033; VC: OR 0.320; 95% CI 0.152–0.674; *p* = 0.003, Supplementary Table S4). Serum EGF levels were significantly lower in patients who had received neoadjuvant chemotherapy in the VC (OR 0.456; 95% CI 0.226–0.922; *p* = 0.029) but not in the TC. The UICC stage did neither influence serum PlGF nor EGF serum levels.

**Table 2:** Univariate analysis of factors associated with serum PlGF and EGF levels.

	Training cohort				Validation cohort			
	PlGF		EGF		PlGF		EGF	
	mean	*p*	mean	*p*	mean	*p*	mean	*p*
**Sex**								
male	16.4	0.100	240.9	0.348	15.5	0.011	328.6	0.261
female	15.1		267.4		13.6		366.0	
**Age (y)**								
≤70	15.5	0.206	272.6	0.022	14.4	0.216	364.6	0.042
>70	16.5		219.4		15.3		305.9	
**BMI [kg/cm** ^**2**^ **]**								
≤25	15.8	0.639	285.7	0.248	13.9	0.035	317.2	0.106
>25	16.3		252.2		15.4		364.9	
**Site of disease**								
Colon	15.7	0.793	267.8	0.229	14.0	0.077	374.6	0.075
Rectum	15.9		234.7		15.3		319.8	
**Validation cohort**								
Yes	17.6	0.016	252.2	0.986	16.9	<0.001	289.3	0.037
No	15.3		251.6		14.0		361.9	
**pT**								
0/Tis/1/2	15.5	0.522	237.3	0.440	14.1	0.165	354.9	0.946
3/4	16.1		261.3		15.1		337.0	
**pN**								
0	16.2	0.510	225.2	0.032	14.6	0.608	354.7	0.552
1/2	15.3		288.1		15.0		320.5	
**UICC stage**								
I/II	16.2	0.356	225.2	0.032	14.6	0.563	355.3	0.307
III	15.3		288.1		15.0		320.5	
**Grade**								
1/2	14.14	0.551	51.65	0.104	15.2	0.831	336.5	0.934
3/4	15.68		253.96		14.5		348.4	

Abbreviations: BMI, body mass index; UICC, Union Internationale Contre le Cancer; PlGF, Phosphatidylinositol-glycan biosynthesis class F protein/Placental growth factor; EGF, epidermal growth factor.

**Table 3:** Multivariate analysis of factors associated with serum PlGF and EGF levels.

	Training cohort				Validation cohort			
	PlGF		EGF		PlGF		EGF	
	OR (95%CI)	*p*	OR (95%CI)	*p*	OR (95%CI)	*p*	OR (95%CI)	*p*
Sex	0.736 (0.397–1.365)	0.331	0.962 (0.480–1.929)	0.906	0.585 (0.297–1.152)	0.121	0.970 (0.503–1.871)	0.948
Age ≤70 [years]	1.699 (0.898–3.214)	0.103	0.571 (0.287–1.138)	0.142	0.425 (0.215–0.840)	0.014	0.899 (0.463–1.745)	0.808
BMI ≤ 25 [kg/cm2]	1.041 (0.557–1.946)	0.901	0.903 (0.458–1.782)	0.769	0.439 (0.226–0.852)	0.015	0.953 (0.879–1.032)	0.234
Site of disease	0.633 (0.329–1.336)	0.250	1.060 (0.541–2.075)	0.771	0.799 (0.369–1.731)	0.570	0.670 (0.318–1.412)	0.292
Neoadjuvant therapy	0.457 (0.222–0.940)	0.033	0.921 (0.365–2.322)	0.861	0.320 (0.152–0.674)	0.003	0.456 (0.226 − 0.922)	0.029
UICC I/II vs III	0.876 (0.462–1.626)	0.656	1.668 (0.843–3.330)	0.142	0.937 (0.470–1.870)	0.880	0.844 (0.435–1.636)	0.597

Abbreviations: BMI, body mass index; UICC, Union Internationale Contre le Cancer; PlGF, Phosphatidylinositol-glycan biosynthesis class F protein/Placental growth factor; EGF, epidermal growth factor.

### Prognostic impact of serum PlGF and EGF levels

#### Overall survival

**Figure 1:**
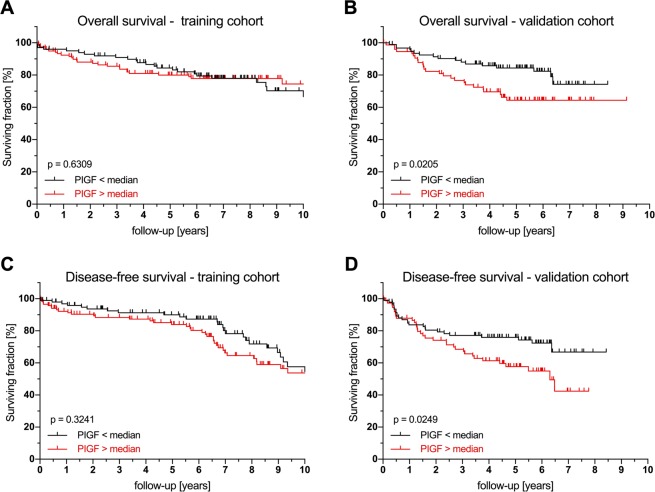
Prognostic effect of serum PlGF levels on overall and disease-free survival in training and validation cohorts. Survival analysis for serum PlGF levels in the training and validation cohorts for overall survival and disease-free survival (log-rank test). Abbreviations: PIGF, Phosphatidylinositol-glycan biosynthesis class F protein/Placental growth factor.

**Figure 2:**
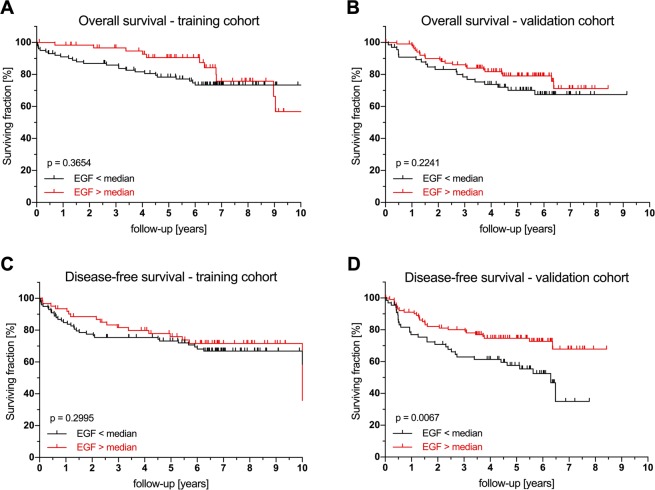
Prognostic effect of serum EGF levels on overall and disease-free survival in training and validation cohorts. Survival analysis for serum EGF levels in the training and validation cohorts for overall survival and disease-free survival (log-rank test). Abbreviations: EGF, epidermal growth factor.

Within the follow-up periods of 6.8 years (+/− 3.9, TC) and 4.7 years (+/− 2.1, VC), 54 patients (24.7%) and 42 patients (25.0%) died, respectively. Of the clinical variables, only age >70 years (TC: *p* = 0.009; VC *p* < 0.001) and UICC III (TC: *p* = 0.065; VC: *p* = 0.013) were significantly associated with impaired OS on univariate survival analysis. Serum PlGF levels correlated significantly with OS in the VC only (TC: *p* = 0.630; VC: *p* = 0.020) when using the median as cutoff (Fig. 1); stronger results were seen when using the 75^th^ percentile as cutoff (Supplementary Fig. S3) (Figs 1,2). EGF (Fig. S2, Supplementary Figs S2 & S4), VEGF, PDGF-A/B, and bFGF serum levels failed to show any significant influence on OS. Additionally, low Ang1 **(***p* = 0.015) and elevated IL-8 (*p* = 0.022) were associated with reduced OS in the VC. In the multivariate Cox regression model, adjusted for age, sex, SOD and UICC stage, only elevated PlGF (>75^th^ percentile) was significantly associated with reduced OS in both cohorts (TC: Hazard ratio (HR): 1.056; 95% CI 1.001–1.114; *p* = 0.046; VC: HR 1.093; 95% CI 1.003–1.191; *p* = 0.001). The data for cutoffs other than the median showed similar results and are reported in Supplementary Figs S1–S4.

#### Disease-free survival

A total of 31 patients (14.2%) in the TC and 37 (22.0%) in the VC developed recurrent disease within the follow-up period. Univariate analysis identified age >70 (TC: *p* = 0.018; VC: *p* < 0.001), UICC stage (TC: *p* = 0.009; VC: *p* = 0.028) and neoadjuvant therapy in the VC (*p* = 0.046), but not in the TC, as prognostic factors for DFS. Of the measured angiogenic factors, serum PlGF levels significantly correlated with DFS in the VC cohort (TC: *p* = 0.324; VC *p* = 0.024) when using the median as cutoff (Fig. 1), when using the 75^th^ percentile as cutoff, significant effects were seen in both cohorts (Supplementary Fig. S3). Similarly, serum EGF levels correlated with DFS significantly in the VC (TC: *p* = 0.299; VC *p* < 0.01, Fig. 2); these effects were even stronger when using the 75^th^ percentile as cutoff (Supplementary Fig. S4). Multivariate analysis showed elevated PlGF to be associated with worse DFS in both cohorts (TC: HR 1.052; 95% CI 1.005–1.107; *p* = 0.029; VC: HR 1.091; 95% CI 1.033–1.153; *p* = 0.009). Conversely, elevated serum EGF significantly predicted longer DFS in both cohorts (TC: HR 0.998; 95% CI 0.996–1.000; *p* = 0.045; VC: HR 0.998; 95% CI 0.996–1.000; *p* = 0.018). Serum VEGF, Ang1, PDGF-A/B, IL-8 and bFGF levels did not show any influence on DFS **(**Table 4**)**. Again, the 75^th^ percentile as cutoff exhibited the strongest effects. The results for other cutoffs are given in Supplementary Figs S1–S4, significance levels for all analyses are reported in Supplementary Table S5. Of note, no synergistic effects of PIGF and EGF were found (Supplementary Table S6) and the inclusion of neoadjuvant therapy in the Cox proportional hazard model does not significantly change the results reported above (Supplementary Table S7).

**Table 4:** Survival analysis of factors associated with serum PlGF and EGF levels.

v	Training cohort			Validation cohort		
	*Log-rank* ^*δ*^	HR (95% CI)^*ε*^	*p* ^*ζ*^	*Log-rank* ^*δ*^	HR (95% CI)^*ε*^	*p* ^*ζ*^
**PlGF**						
OS	0.003*	1.056 (1.001–1.114)	0.046	0.021*	1.093 (1.003–1.191)	0.001
DFS	0.035*	1.052 (1.005–1.107)	0.029	<0.001*	1.091 (1.033–1.153)	0.009
**EGF**						
OS	0.162*		0.201	0.400*		0.453
DFS	0.027	0.998 (0.996–1.0)	0.045	0.065*	0.998 (0.996–1.000)	0.018
**VEGF**						
OS	0.328*		0.087	0.360*		0.368
DFS	0.350*		0.153	0.344*		0.376
Validation cohort						
OS	0.745*		0.978	0.015*		0.140
DFS	0.699*		0.793	0.056*		0.413
**PDGF-A**						
OS	0.807*		0.370	0.101*		0.062
DFS	0.286*		0.575	0.278*		0.116
**PDGF-B**						
OS	0.647*		0.808	0.534*		0.183
DFS	0.728*		0.993	0.987*		0.375
**IL-8**						
OS	0.393*		0.671	0.022*		0.564
DFS	0.838*		0.652	0.064*		0.617
**bFGF**						
OS	0.190*		0.399	0.855*		0.470
DFS	0.691*		0.948	0.564*		0.824
**Sex**						
OS	0.386		0.239	0.692		0.932
DFS	0.136		0.064	0.481		0.811
**Age >70**						
OS	0.009		0.365	<0.001	4.3 (2.2–8.3)	<0.001
DFS	0.018		0.628	<0.001	2.2 (1.3–3.8)	0.004
**SOD**						
OS	0.985		—	0.752		—
DFS	0.311		—	0.546		—
**neoadj. Tx**						
OS	0.759		—	0.510		—
DFS	0.155		—	0.046		0.140
**pT**						
OS	0.638		—	0.001		—
DFS	0.087		—	0.004		—
**pN**						
OS	0.064		—	0.015		—
DFS	0.009		—	0.031		—
**UICC stage**						
OS	0.064	2.2 (1.1–4.3)	0.020	0.013	1.7 (0.9–3.9)	0.086
DFS	0.009	2.8 (1.5–5.0)	0.001	0.028	1.9 (1.1–3.4)	0.022
**Grade**						
OS	0.188		—	0.506		—
DFS	0.338		—	0.677		—

^δ^P-values using the univariate log-rank test, median was used as a cutoff for PlGF and EGF; ^ε^Hazard ratio (HR) and 95% confidence interval (CI) for the multivariate Cox regression model; ^ζ^p-values of the multivariate Cox regressions model; *75%-percentile was used as a cutoff.

Abbreviations: BMI, body mass index; UICC, Union Internationale Contre le Cancer; PlGF, Phosphatidylinositol-glycan biosynthesis class F protein/placental growth factor; EGF, epidermal growth factor; SOD, site of disease; OS, overall survival; PFS, progression free survival.

### Prognostic impact of intratumoral PlGF and EGF expression

**Figure 3:**
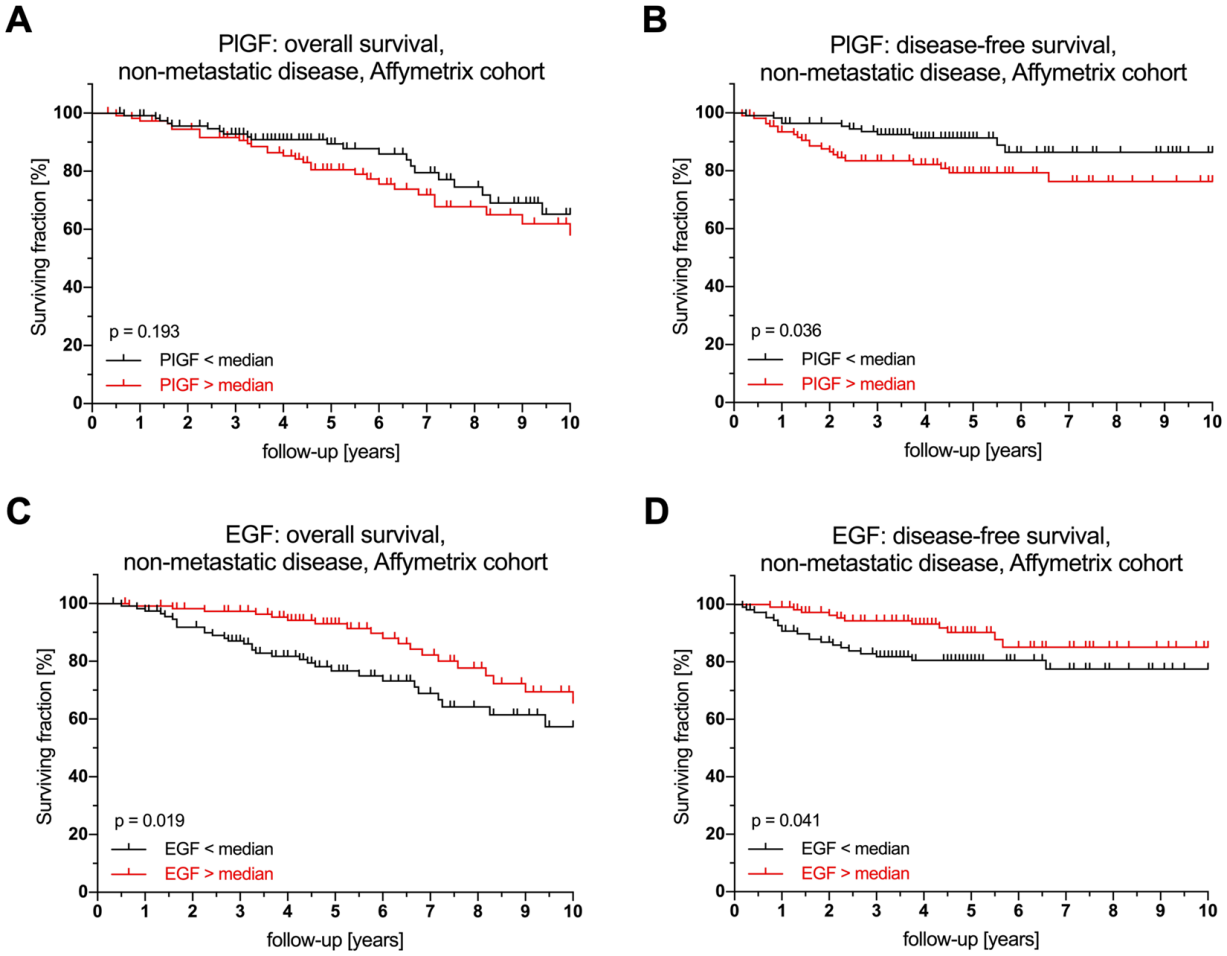
Prognostic effect of PlGF and EGF RNA expression on overall and disease-free survival in non-metastatic disease in the Affymetrix microarray cohort. Survival analysis for PlGF and EGF expression levels in non-metastatic disease in the Affymetrix cohort for overall survival and disease-free survival (log-rank test). Survival analyses with different cutoffs are displayed in Supplementary Fig. S6. Abbreviations: PIGF, Phosphatidylinositol-glycan biosynthesis class F protein/Placental growth factor; EGF, epidermal growth factor.

Given the prognostic relevance of serum PlGF (OS + DFS) and EGF (DFS), we hypothesized that these cytokines may be produced by tumor cells and thus analyzed their prognostic role in two publically available, independent cohorts: The Affymetrix cohort with 550 CRC patients of all stages and expression data from Affymetrix microarrays^24^, and the TCGA cohort with 463 CRC patients of all stages and RNA expression data generated via RNAseq^25^. In both cohorts, our findings were independently confirmed (Fig. 3).

There was no influence of EGFR expression levels on the prognostic value of EGF in both cohorts (Supplementary Fig. S5). In patients with non-metastatic disease in the Affymetrix cohort, PlGF expression higher than the median showed a tendency towards being a negative prognostic factor for overall survival, but failed to reach statistical significance (p = 0.193; HR 1.45; 95% CI 0.83–2.55). In the same patients, EGF proved to be a positive prognostic factor for overall survival (p = 0.019; HR 0.51; 95% CI 0.29–0.9). For disease-free survival, both factors were confirmed as negative (PlGF, p = 0.036; HR 2.14; 95% CI 1.03–4.45) and positive (EGF, p = 0.041; HR 0.48; 95% CI 0.23–0.99) prognostic factors. There was no data on disease recurrence available for the TCGA cohort.

**Figure 4:**
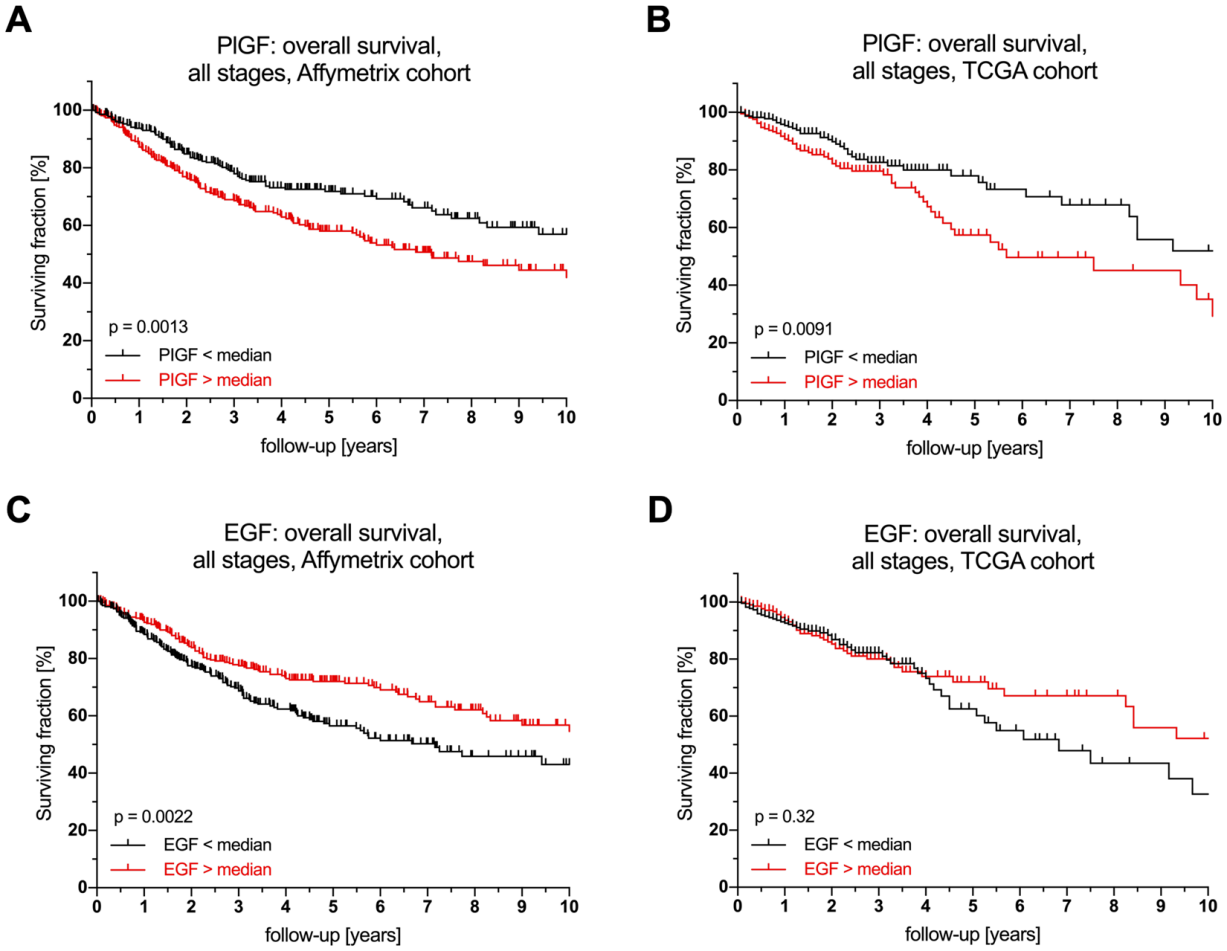
Prognostic effect of PlGF and EGF RNA expression on overall survival in patients of all stages in the Affymetrix microarray and TCGA RNAseq cohorts. Survival analysis for PlGF and EGF expression levels in patients of all stages in the Affymetrix and TCGA cohorts for overall survival (log-rank test). Survival analyses with different cutoffs are displayed in Supplementary Fig. S7. Abbreviations: PIGF, Phosphatidylinositol-glycan biosynthesis class F protein/Placental growth factor; EGF, epidermal growth factor; TCGA, The Cancer Genome Atlas.

When including metastatic patients, PlGF and EGF expression again showed the same prognostic effects as demonstrated before: While PlGF was a negative prognostic factor for overall survival in both the Affymetrix and the TCGA cohorts (Affymetrix cohort: p = 0.0013; HR 1.59; 95% CI 1.2–2.11; TCGA cohort: p = 0.0091; HR 1.74; 95% CI 1.14–2.64), EGF was a significant positive prognostic factor for overall survival in the Affymetrix cohort (p = 0.0022; HR 0.65; 95% CI 0.49–0.86) und but not in the TCGA cohort (p = 0.32; HR 0.81; 95% CI 0.54–1.23) (Fig. 4).

## Discussion

This study demonstrates elevated serum PlGF levels to be a negative prognostic factor for OS and DFS in non-metastatic CRC patients. This prognostic association was demonstrated in two independent patient cohorts. Additionally, elevated serum EGF levels are associated with favorable DFS, but not OS, in both cohorts. Serum VEGF, Ang1, PDGF-A/B, IL-8 and bFGF levels did not predict OS or DFS on multivariate analysis. Our findings suggest that quantification of serum PlGF and EGF may be useful for preoperative risk stratification of patients with non-metastatic CRC. On tissue RNA level, identical prognostic effects could be demonstrated for both cytokines in two other independent cohorts with expression data measured via microarray and RNA next generation sequencing, respectively, thus strongly confirming the data from our own cohorts.

The currently available body of evidence on PlGF and EGF in CRC is limited. Gomceli *et al*. reported high PlGF to be prognostic for local recurrence but not OS in non-metastatic CRC, thus supporting our findings^29^. Contradictory to our present finding of PlGF as a negative prognostic marker, previously published data from our own group demonstrated PlGF to be of positive prognostic value for recurrence-free survival in metastatic CRC^30^. However, different cutoffs were chosen in the metastatic cohort previously published and only patients with PlGF levels >90^th^ percentile experienced favorable survival. Additionally, PlGF indicated only favorable disease-free survival and was not associated with overall survival. Moreover, the previously published data are from metastatic patients undergoing liver resection for CRC metastases, representing a different patient cohort as well as a different microenvironment in hepatic metastases. These differences in the angiogenic microenvironment between hepatic metastases and the primary colorectal tumor have resulted in anti-VEGF treatment to be used only in metastatic disease and may thus also explain the different effects of PlGF in different disease stages. This disparity in tumor biology is emphasized by data indicating that primary tumor cell lines produced more PlGF than cell lines from metastatic CRC^31^.

PlGF stimulates the chemotactic migration of human mesenchymal progenitor cells and stimulates neovascularization via the VEGF pathway, especially under hypoxic conditions^32,33^, and facilitates vessel growth and maturation^34^. PlGF is able to independently activate endothelial cells and synergizes with VEGF in driving angiogenesis^12,35^. In addition to angiogenesis, PlGF influences intratumoral macrophage polarization towards an immunosuppressive phenotype via the VEGF1 pathway^36^. Furthermore, PlGF, but not VEGF-A or VEGF-B, is increased in obesity^15,36^. Our data confirmed a correlation of PlGF levels with obesity and may partly explain the clinically well-known unfavorable outcome of obese cancer patients^15,36^. While the data presented here is insufficient to confirm PlGF as the reason for the more malignant biology of cancer in obese patients, they clearly warrant further studies on this matter.

An interesting finding is the markedly increased PlGF level in patients after neoadjuvant therapy. The addition of bevacizumab, a humanized, monoclonal antibody targeting VEGF-A, to chemoradiation in the neoadjuvant setting for non-metastatic rectal cancer has limited benefit, which may indicate escape mechanisms to bevacizumab treatment^37,38^. As PlGF is elevated during neoadjuvant therapy and able to activate the VEGF signaling axis independent of VEGF-A or -B^39^, it may act as a mediator of such escape mechanisms after bevacizumab-mediated VEGF depletion. This theory is supported by the activity of PlGF inhibitors in anti-VEGF-resistant tumors^40,41^. Consequently, elevated PlGF may be a negative predictive factor in this setting and if so, agents targeting VEGF signaling downstream of PlGF may be of more benefit than bevacizumab. This question needs to be addressed in future clinical trials investigating neoadjuvant anti-angiogenic therapy in rectal cancer.

In addition to PlGF, our data identified elevated serum EGF levels as a positive prognostic factor in CRC. The biological role of EGF in CRC is currently unclear. Although intuitively it should act as a negative prognostic factor due to its activation of the MAPK/RAS/RAF signaling cascade, previous studies were unable to demonstrate a correlation between serum EGF levels and prognosis in CRC^21^ and the here presented study even observed a positive correlation. In breast cancer, the prognostic value of EGF is also a matter of debate while most studies point toward a positive correlation between serum EGF levels and prognosis^42,43^. The EGF receptor (EGFR, HER1, ErbB1) is a receptor for ligands of the epidermal growth factor family. Other receptors in this family include HER2 (ErbB2), HER3 (ErbB3) and HER4 (ErbB4)^44^. Upon ligand binding, receptors of the HER family form homo- or heterodimers, thus potentiating the MAPK activation as compared to their activity as monomers^44^. Ligands of the EGF family include EGF, Heparin-binding EGF-like growth factor (HB-EGF), transforming growth factor-α (TGF-α), Amphiregulin (AR), Epiregulin (EPR), Epigen, Betacellulin (BTC), neuregulin-1 (NRG1), neuregulin-2 (NRG2), neuregulin-3 (NRG3) and neuregulin-4 (NRG4)^45^. While EGFR is activated by EGF, HB-EGF, TFG-α, AR, EPR, BTC and Epigen, neuregulins activate HER3 and HER4 and no ligand is known for HER2^44^, suggesting a role of HER2 as a co-receptor transactivated by EGFR ligands. In summary, the EGF ligand and receptor families form a complex and incompletely understood network. The reason for the positive prognostic role of EGF is still unknown and leaves room for speculation: Dropping EGF serum levels after resection of CRC suggest the origin of elevated EGF levels within the tumor^46^. Low intratumoral MAPK/RAS/RAF signaling activity, suggesting low proliferative activity of the tumor cells and thus favorable prognosis, may lead to compensatory EGF excretion in tumor cells. Interestingly, EGF seems to be a prognostic factor in CRC independently of both activating RAS/RAF mutations and cetuximab treatment^47^, suggesting a role of EGF outside of the MAPK/RAS/RAF axis, possibly adding a second explanatory approach. Clearly, further research investigating the role of EGF in CRC is needed.

In this study, we compared the prognostic effects of serum proteins in some cohorts (TC and VC) and the prognostic effects of the respective mRNA levels in other cohorts. The correlation between mRNA and protein levels is highly dependend on the stability of both mRNA and protein. While in steady state mRNA the concordance between mRNA and protein abundance is 100%, the concordance between mRNA and protein variation is significantly lower^48^. Despite this, we found highly concordant prognostic effects between groups with protein and mRNA data; however, the comparison between mRNA and protein levels can still be considered a limitation of this study.

The here presented study includes a prospective training cohort and an independent prospective validation cohort which confirmed the main findings of the training cohort, thus representing high quality evidence. In addition, most findings could be validated in two more large cohorts, thus adding to the high level of evidence. The conclusions are clinically relevant, but the underlying molecular biology is still poorly understood. Therefore, molecular studies investigating the mechanistic principles as well as prospective clinical trials involving different centers and patient collectives are required prior to implementation of PlGF and EGF serum testing in clinical routine.

In summary, this study suggests preoperative serum PlGF and EGF levels as prognostic factors in non-metastatic CRC. The prognostic value of these cytokines in two independent prospective and another two independent retrospective cohorts supports their evaluation in larger multi-center studies on preoperative risk stratification and may ultimately enable clinicians to identify subgroups of patients who may benefit from adjuvant therapy while sparing others the side effects of potentially unnecessary systemic treatment.

## Supplementary information

Supplementary Dataset 1

## Data Availability

All authors declare that they had full access to all information and data published in this article. These data have not been published elsewhere.
