# Supplementary material for: Serum PlGF and EGF are independent prognostic markers in non-metastatic colorectal cancer
Source: Sci Rep. 2019 Jul 29;9:10921. doi: 10.1038/s41598-019-47429-5 (PMC6662856; doi:10.1038/s41598-019-47429-5)
Supplement: Supplementary file 1 — Supplementary Dataset 1 [file 41598_2019_47429_MOESM1_ESM.docx]

**Serum PlGF and EGF are independent prognostic markers in non-metastatic colorectal cancer**

Sebastian Schölch^1,2,3,4^*, Andreas Bogner^2^*, Ulrich Bork^2^, Mohammad Rahbari^2^, Balázs Győrffy^5,6^, Martin Schneider^3,4,7^, Christoph Reissfelder^1,2,3,4^, Jürgen Weitz^2,3,4^ & Nuh N. Rahbari^1,2^

1. Department of Surgery, Universitätsmedizin Mann­heim, Medical Faculty Mannheim, Heidelberg University, Mannheim, Germany
2. Department of Gastrointestinal, Thoracic and Vascular Surgery, Medizinische Fakultät *Carl Gustav Carus*, Technische Universität Dresden, Dresden, Germany
3. German Cancer Consortium
4. German Cancer Research Center (DKFZ), Heidelberg, Germany
5. MTA TTK Lendület Cancer Biomarker Research Group, Magyar Tudósok körútja 2., H-1117, Budapest, Hungary
6. Semmelweis University, 2nd Department of Pediatrics, Bókay u. 53-54., H-1083, Budapest, Hungary
7. Department of General, Gastrointestinal and Transplant Surgery, University Hospital Heidelberg, Medical Faculty Heidelberg, Heidelberg University, Heidelberg, Germany

** Sebastian Schölch and Andreas Bogner contributed equally to this work.*

**Editorial correspondence:**

Sebastian Schölch

Department of Surgery

Medizinische Fakultät Mannheim

Ruprecht-Karls-Universität Heidelberg

Theodor-Kutzer-Ufer 1-3, 68167 Mannheim, Germany

E-Mail: sebastian.schoelch@umm.de

Phone: +49-621-383-2225

**SUPPLEMENTARY TABLES**

**Supplementary Table S1.** Arithmetic mean and Standard deviation of serum VEGF, Ang1, PDGFA/B, IL-8 and bFGF levels in the training and validation cohorts.

|  | **VEGF** | **Ang1** | **PDGFA** | **PDGFB** | **IL-8** | **bFGF** |
| --- | --- | --- | --- | --- | --- | --- |
| **Training cohort** |  |  |  |  |  |  |
| *Mean (pg/ml)* | 279.7 | 64 791.6 | 2 148.4 | 2 117.5 | 20.1 | 4.2 |
| *Standard deviation* | 259.5 | 12 625.9 | 1 242.7 | 1 408.1 | 27.6 | 4.5 |
| **Validation cohort** |  |  |  |  |  |  |
| *Mean (pg/ml)* | 324.6 | 38 379.2 | 2 854.3 | 2 821.4 | 22.5 | 5.4 |
| *Standard deviation* | 235.7 | 20 280.9 | 1 001.9 | 1 116.7 | 17.7 | 8.7 |

|  | **Training cohort** | | | | | |  |  |  |
| --- | --- | --- | --- | --- | --- | --- | --- | --- | --- |
|  | **VEGF** | **Ang1** | | **PDGF-A** | **PDGF-B** | | **IL-8** | **bFGF** | |
|  | *p* | | *p* | *p* | | *p* | *p* | | *p* |
| **Sex** |  | |  |  | |  |  | |  |
| male | 0.073 | | 0.303 | 0.087 | | 0.111 | 0.238 | | 0.975 |
| female |  |  |  |  |  |  |  |  |  |
| **Age (y)** |  | |  |  | |  |  | |  |
| ≤70 | 0.057 | | 0.284 | 0.563 | | 0.363 | 0.605 | | 0.284 |
| >70 |  |  |  |  |  |  |  |  |  |
| **BMI [kg/cm2]** |  | |  |  | |  |  | |  |
| ≤25 | 0.123 | | 0.311 | 0.127 | | 0.506 | 0.088 | | 0.483 |
| >25 |  |  |  |  |  |  |  |  |  |
| **Site of disease** |  | |  |  | |  |  | |  |
| Colon | **0.012** | | **0.010** | **0.040** | | **0.001** | 0.562 | | 0.333 |
| Rectum |  |  |  |  |  |  |  |  |  |
| **Neoadjuvant therapy** |  | |  |  | |  |  | |  |
| Yes | 0.761 | | 0.667 | 0.211 | | 0.370 | 0.436 | | 0.306 |
| No |  |  |  |  |  |  |  |  |  |
| **pT** |  | |  |  | |  |  | |  |
| 0/Tis/1/2 | 0.568 | | 0.762 | 0.700 | | 0.886 | <0.001 | | 0.166 |
| 3/4 |  |  |  |  |  |  |  |  |  |
| **pN** |  | |  |  | |  |  | |  |
| 0 | 0.686 | | 0.095 | 0.803 | | 0.612 | 0.638 | | 0.680 |
| 1/2 |  |  |  |  |  |  |  |  |  |
| **UICC stage** |  | |  |  | |  |  | |  |
| I/II | 0.470 | | 0.095 | 0.362 | | 0.597 | 0.138 | | 0.401 |
| III |  |  |  |  |  |  |  |  |  |
| **Grade** |  | |  |  | |  |  | |  |
| 1/2 | 0.077 | | 0.164 | 0.159 | | 0.160 | 0.309 | | 0.411 |
| 3/4 |  |  |  |  |  |  |  |  |  |

**Supplementary Table S2.** Correlation of clinicopathological characteristics with serum VEGF, Ang1, PDGF-A/B, IL-8 and bFGF levels in the training cohort.

Abbreviations: BMI, body mass index; VEGF, vascular endothelial growth factor; Ang1, angiopoetin 1; PDGF-A/B, platelet-derived growth factor A/B; IL-8, interleukin 8; bFGF, basic fibroblast growth factor; UICC, Union Internationale Contre le Cancer.

**Supplementary Table S3.** Correlation of clinicopathological characteristics with serum VEGF, Ang1, PDGF-A/B, IL-8 and bFGF levels in the validation cohort.

Abbreviations: BMI, body mass index; VEGF, vascular endothelial growth factor; Ang1, angiopoetin 1; PDGF-A/B, platelet-derived growth factor A/B; IL-8, interleukin 8; bFGF, basic fibroblast growth factor; UICC, Union Internationale Contre le Cancer.

|  | **Validation cohort** | | | | | |  |  |  |
| --- | --- | --- | --- | --- | --- | --- | --- | --- | --- |
|  | **VEGF** | **Ang1** | | **PDGF-A** | **PDGF-B** | | **IL-8** | **bFGF** | |
|  | *p* | | *p* | *p* | | *p* | *p* | | *p* |
| **Sex** |  | |  |  | |  |  | |  |
| male | 0.803 | | **0.002** | 0.225 | | 0.652 | 0.339 | | 0.332 |
| female |  |  |  |  |  |  |  |  |  |
| **Age (y)** |  | |  |  | |  |  | |  |
| ≤70 | 0.622 | | 0.446 | 0.152 | | 0.901 | 0.313 | | 0.328 |
| >70 |  |  |  |  |  |  |  |  |  |
| **BMI [kg/cm2]** |  | |  |  | |  |  | |  |
| ≤25 | 0.114 | | 0.297 | 0.334 | | 0.117 | 0.300 | | 0.181 |
| >25 |  |  |  |  |  |  |  |  |  |
| **Site of disease** |  | |  |  | |  |  | |  |
| Colon | 0.328 | | **<0.001** | 0.224 | | 0.316 | 0.213 | | 0.745 |
| Rectum |  |  |  |  |  |  |  |  |  |
| **Neoadjuvant therapy** |  | |  |  | |  |  | |  |
| Yes | 0.203 | | **<0.001** | 0.334 | | 0.351 | 0.936 | | **0.005** |
| No |  |  |  |  |  |  |  |  |  |
| **pT** |  | |  |  | |  |  | |  |
| 0/Tis/1/2 | 0.629 | | 0.442 | 0.646 | | 0.758 | **<0.001** | | 0.648 |
| 3/4 |  |  |  |  |  |  |  |  |  |
| **pN** |  | |  |  | |  |  | |  |
| 0 | 0.449 | | 0.239 | 0.625 | | 0.491 | 0.461 | | 0.133 |
| 1/2 |  |  |  |  |  |  |  |  |  |
| **UICC stage** |  | |  |  | |  |  | |  |
| I/II | 0.433 | | 0.403 | 0.636 | | 0.662 | 0.052 | | 0.102 |
| III |  |  |  |  |  |  |  |  |  |
| **Grade** |  | |  |  | |  |  | |  |
| 1/2 | 0.246 | | 0.816 | 0.179 | | 0.153 | 0.687 | | 0.595 |
| 3/4 |  |  |  |  |  |  |  |  |  |

**Supplementary Table S4.** Arithmetic mean and standard deviation of serum PlGF and EGF in patients with or without neoadjuvant therapy in the training and validation cohorts including survival analysis

Abbreviations: PlGF, Phosphatidylinositol-glycan biosynthesis class F protein/placental growth factor; EGF, Epidermal growth factor; neoadj., neoadjuvant; DFS, disease free survival; *log rank testing was performed; survival time is given in years, mean (Standard deviation), 75% cutoff was used for analyses.

|  | **PlGF** | | | **EGF** | | |
| --- | --- | --- | --- | --- | --- | --- |
|  | **Neaoadj.** | **No neoadj.** | ***p*-value** | **Neaoadj.** | **No neoadj.** | ***p*-value** |
| **Training cohort** |  |  |  |  |  |  |
| *Mean (SD) pg/ml* | 17.6 (6.4) | 15.3 (5.5) | **0.029** | 252.2 (153.1) | 251.6 (182.7) | 0.985 |
| Overall survival* | 5.8 (3.0) | 7.1 (4.0) | **0.022** |  |  |  |
| DFS* | 4.8 (3.2) | 6.8 (4.2) | 0.155 |  |  |  |
| **Validation cohort** |  |  |  |  |  |  |
| *Mean (SD) pg/ml* | 16.9 (4.2) | 14.0 (4.4) | **<0.001** | 289.3 (156.4) | 361.9 (206.7) | 0.018 |
| Overall survival* | 4.9 (2.2) | 4.6 (2.0) | 0.510 |  |  |  |
| DFS* | 3.8 (2.3) | 4.2 (2.2) | **0.046** |  |  |  |

**Supplementary Table S5.**

Significance levels of all reported survival analyses (log rank test) with different cutoff levels.

Abbreviations: PlGF, Phosphatidylinositol-glycan biosynthesis class F protein/placental growth factor; EGF, Epidermal growth factor; neoadj., neoadjuvant; DFS, disease free survival; PFS, progression free survival.

|  |  | **Training Cohort** | **Validation Cohort** |
| --- | --- | --- | --- |
|  | **PIGF** | p-value | p-value |
| **DFS/PFS** | 25th percentile | 0.0636 | 0.0551 |
|  | 50th percentile | 0.3241 | **0.0249** |
|  | 75th percentile | **0.0345** | **0.0091** |
| **OS** | 25th percentile | 0.1539 | 0.3147 |
|  | 50th percentile | 0.6309 | **0.0205** |
|  | 75th percentile | **0.0224** | 0.0613 |
|  | **EGF** |  |  |
| **DFS/PFS** | 25th percentile | **0.0072** | **0.0252** |
|  | 50th percentile | 0.2995 | **0.0067** |
|  | 75th percentile | **0.0264** | 0.0653 |
| **OS** | 25th percentile | 0.1 | 0.3241 |
|  | 50th percentile | 0.3654 | 0.2241 |
|  | 75th percentile | 0.1617 | 0.3957 |

**Supplementary Table S6: Analyses of synergistic prognostic effects of PIGF and VEGF**

**δ: p values using the univariate log-rank test, the median was used as a cutoff for PlGF and VEGF ε: Hazard ratio (HR) and 95% confidence interval (CI) for the multivariate Cox regression model; ζ: p-values of the multivariate Cox regressions model;** ↑ high serum levels of PlGF or EGF; ↓ low serum levels of PlGF or EGF.

**Abbreviations:** PlGF, Phosphatidylinositol-glycan biosynthesis class F protein/placental growth factor; VEGF, vascular endothelial growth factor; OS, overall survival; PFS, progression free survival; N/A not available = multivariate analysis was not performed due to insignificant results in the univariate analysis

|  | **Training cohort** | | | **Validation cohort** | | |
| --- | --- | --- | --- | --- | --- | --- |
|  | *Log-rank^δ^* | HR (95% CI)*^ε^* | *P^ζ^* | *Log-rank^δ^* | HR (95% CI)*^ε^* | *P^ζ^* |
| **PlGF*↑* / VEGF*↑*** |  |  |  |  |  |  |
| OS | 0.379 | N/A | N/A | **0.030** | 1.451 (0.774 – 2.718) | 0.246 |
| DFS | 0.259 | N/A | N/A | 0.131 | N/A | N/A |
| **PlGF↓/VEGF↑** |  |  |  |  |  |  |
| OS | 0.388 | N/A | N/A | 0.069 | 0.552 (0.245 - 1.246) | 0.153 |
| DFS | 0.612 | N/A | N/A | 0.327 | N/A | N/A |
| **PIGF*↑* VEGF↓** |  |  |  |  |  |  |
| OS | 0.804 | N/A | N/A | 0.599 | N/A | N/A |
| DFS | 0.878 | N/A | N/A | 0.232 | N/A | N/A |
| **PIGF↓VEGF↓** |  |  |  |  |  |  |
| OS | 0.244 | N/A | N/A | 0.486 | N/A | N/A |
| DFS | 0.220 | N/A | N/A | 0.144 | N/A | N/A |

**Supplementary Table S7: Results of Cox regression analysis adjusted for age, sex, Site of disease (SOD), UICC and neoadjuvant therapy.**

**δ: P-values using the univariate log-rank test, median was used as a cutoff for PlGF and EGF; ε: Hazard ratio (HR) and 95% confidence interval (CI) for the multivariate Cox regression model; ζ: p-values of the multivariate Cox regressions model; * 75%-percentile was used as a cutoff.**

**Abbreviations:** BMI, body mass index; UICC, Union Internationale Contre le Cancer; PlGF, Phosphatidylinositol-glycan biosynthesis class F protein/placental growth factor; EGF, epidermal growth factor; SOD, site of disease; OS, overall survival; PFS, progression free survival.

|  | **Training cohort** | | | **Validation cohort** | | |
| --- | --- | --- | --- | --- | --- | --- |
|  | *Log-rank^δ^* | HR (95% CI)*^ε^* | *P^ζ^* | *Log-rank^δ^* | HR (95% CI)*^ε^* | *P^ζ^* |
| **PlGF** |  |  |  |  |  |  |
| OS | **0.630*** | 1.061 (1.007 - 1.118) | **0.025** | 0.020* | 1.095 (1.030 - 1.195) | **0.004** |
| DFS | **0.324*** | 1.052 (1.005 - 1.107) | **0.029** | **0.025*** | 1.091 (1.031 - 1.148) | **0.002** |
| **EGF** |  |  |  |  |  |  |
| OS | 0.162* |  | 0.072 | 0.400* |  | 0.453 |
| DFS | **0.027** | 0.998 (0.996 - 1.000) | **0.045** | 0.065* | 0.998 (0.996 – 9.999) | **0.011** |
| **Sex** |  |  |  |  |  |  |
| OS | 0.386 |  | 0.287 | 0.692 |  | 0.902 |
| DFS | 0.136 |  | 0.064 | 0.481 |  | 0.933 |
| **Age >70** |  |  |  |  |  |  |
| OS | 0.009 |  | 0.313 | **<0.001** | 4.2 (2.2 - 8.1) | **<0.001** |
| DFS | 0.018 |  | 0.649 | **<0.001** | 2.3 (1.4 – 3.9) | **0.002** |
| **SOD** |  |  |  |  |  |  |
| OS | 0.985 |  | 0.761 | 0.752 |  | 0.560 |
| DFS | 0.311 |  | 0.452 | 0.546 |  | 0.890 |
| **neoadj. Tx** |  |  |  |  |  |  |
| OS | 0.759 |  | 0.897 | 0.510 |  | 0.799 |
| DFS | 0.155 |  | 0.461 | 0.046 |  | 0.487 |
| **UICC stage** |  |  |  |  |  |  |
| OS | 0.064 | 2.2 (1.1 - 4.3) | **0.020** | 0.013 | 1.95 (1.1 - 3.6) | **0.031** |
| DFS | 0.009 | 2.8 (1.5 - 5.0) | **0.001** | 0.028 | 1.8 (1.06 – 3.01) | **0.029** |

**SUPPLEMENTARY FIGURES**


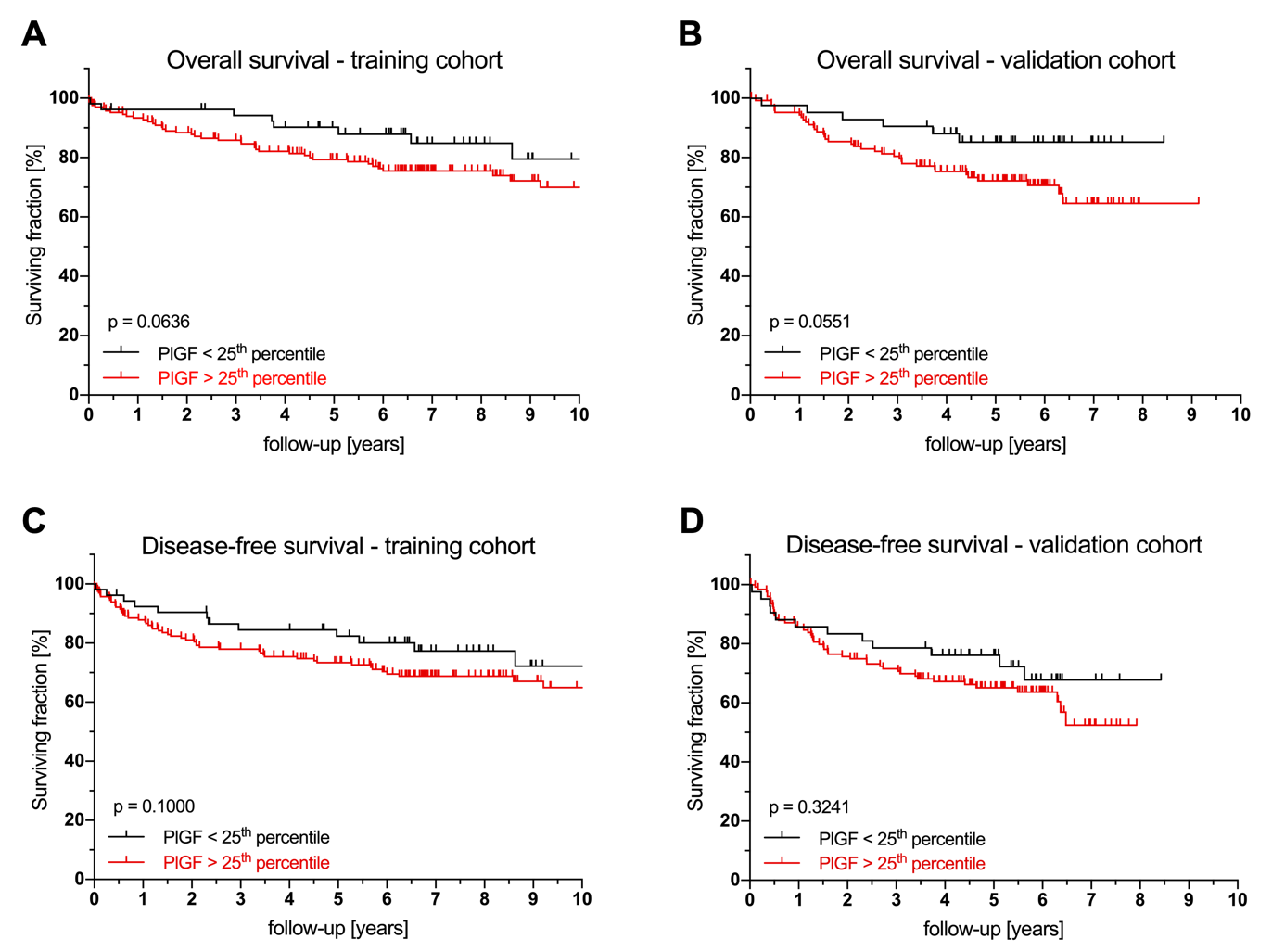


**Supplementary Figure S1: Kaplan Meier curves of serum PlGF levels displaying the univariate survival analysis using the 25^th^ percentile as cutoff.**

Survival analysis for serum PlGF levels in the training and validation cohorts is shown for overall survival and disease-free survival in panels A-D (log-rank test). Abbreviations: PIGF, Phosphatidylinositol-glycan biosynthesis class F protein/Placental growth factor.


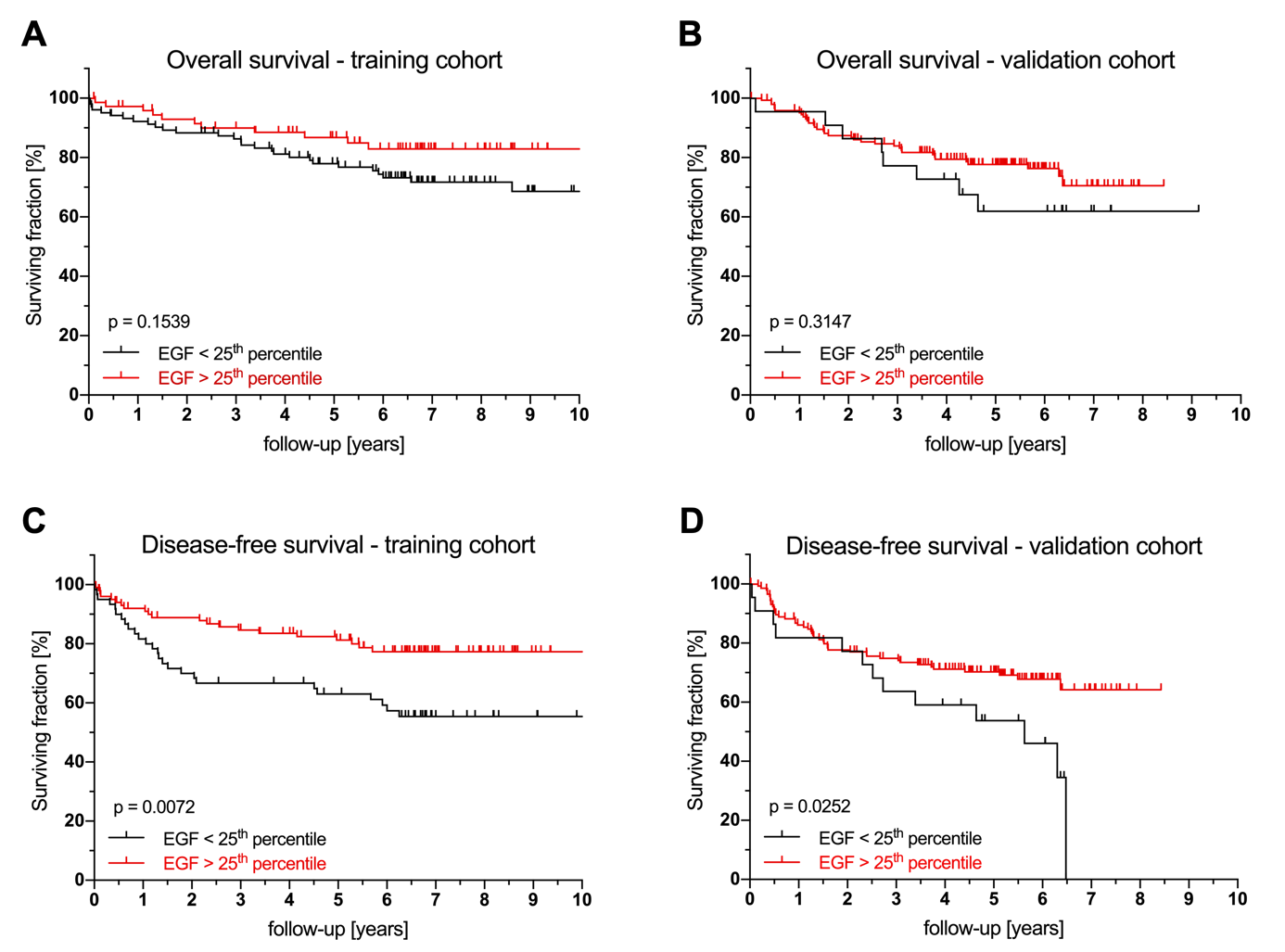


**Supplementary Figure S2: Kaplan Meier curves of serum EGF levels displaying the univariate survival analysis using the 25^th^ percentile as cutoff.**

Survival analysis for serum EGF levels in the training and validation cohorts is shown for overall survival and disease-free survival in panels A-D (log-rank test). Abbreviations: EGF, epidermal growth factor.


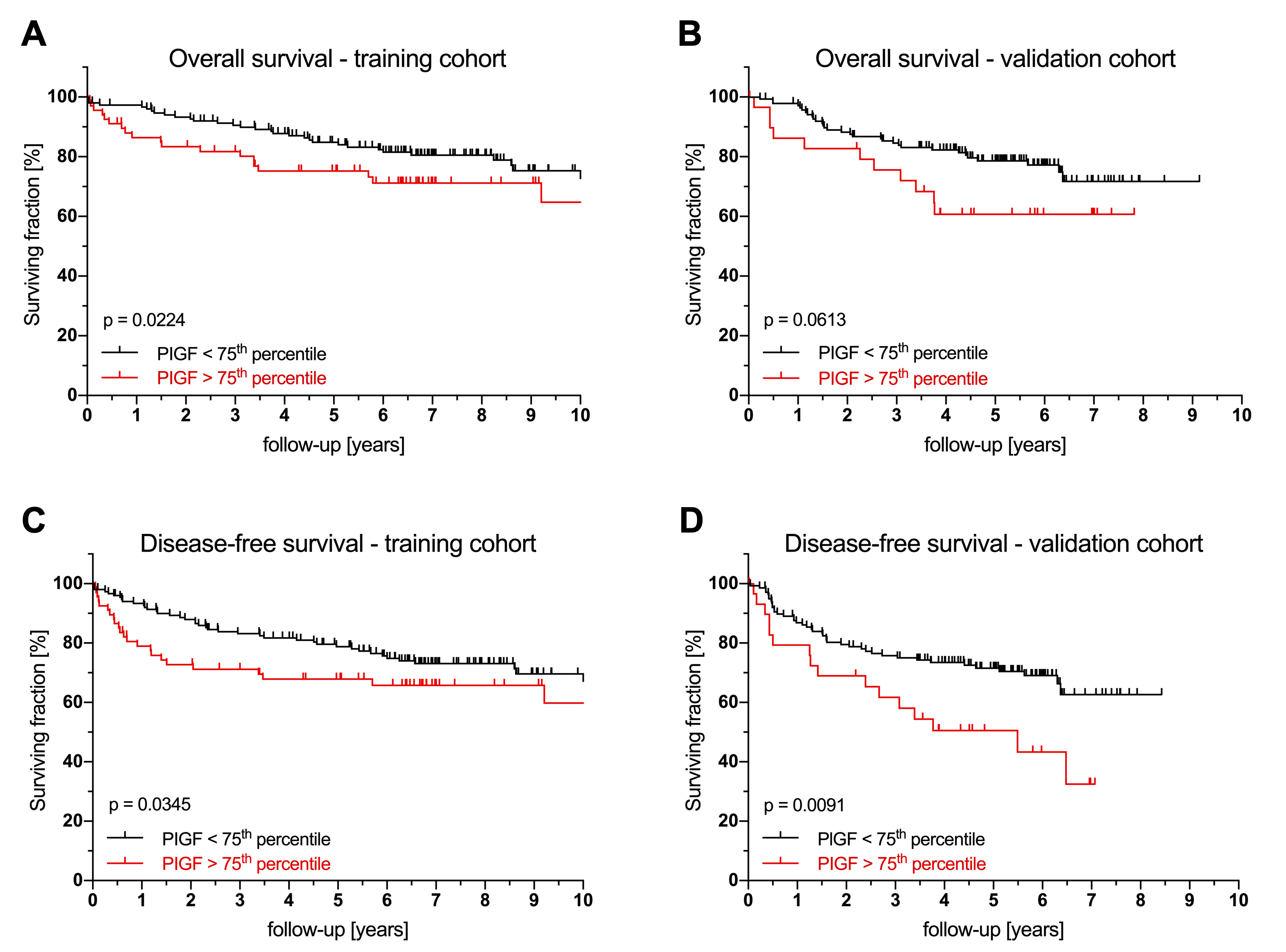


**Supplementary Figure S3: Kaplan Meier curves of serum PlGF levels displaying the univariate survival analysis using the 75^th^ percentile as cutoff.**

Survival analysis for serum PlGF levels in the training and validation cohorts is shown for overall survival and disease-free survival in panels A-D (log-rank test). Abbreviations: PIGF, Phosphatidylinositol-glycan biosynthesis class F protein/Placental growth factor.


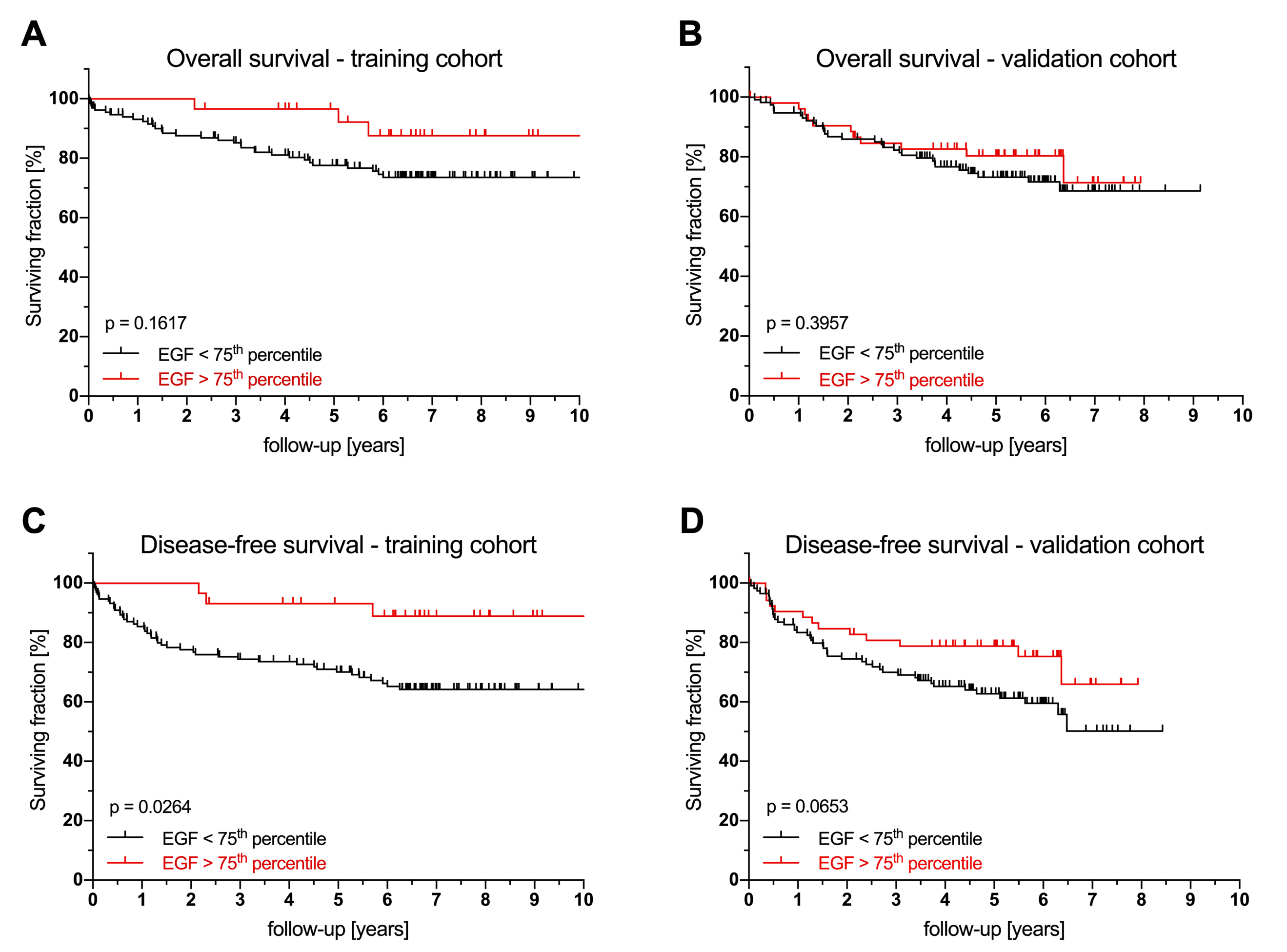


**Supplementary Figure S4: Kaplan Meier curves of serum EGF levels displaying the univariate survival analysis using the 7^th^ percentile as cutoff.**

Survival analysis for serum EGF levels in the training and validation cohorts is shown for overall survival and disease-free survival in panels A-D (log-rank test). Abbreviations: EGF, epidermal growth factor.


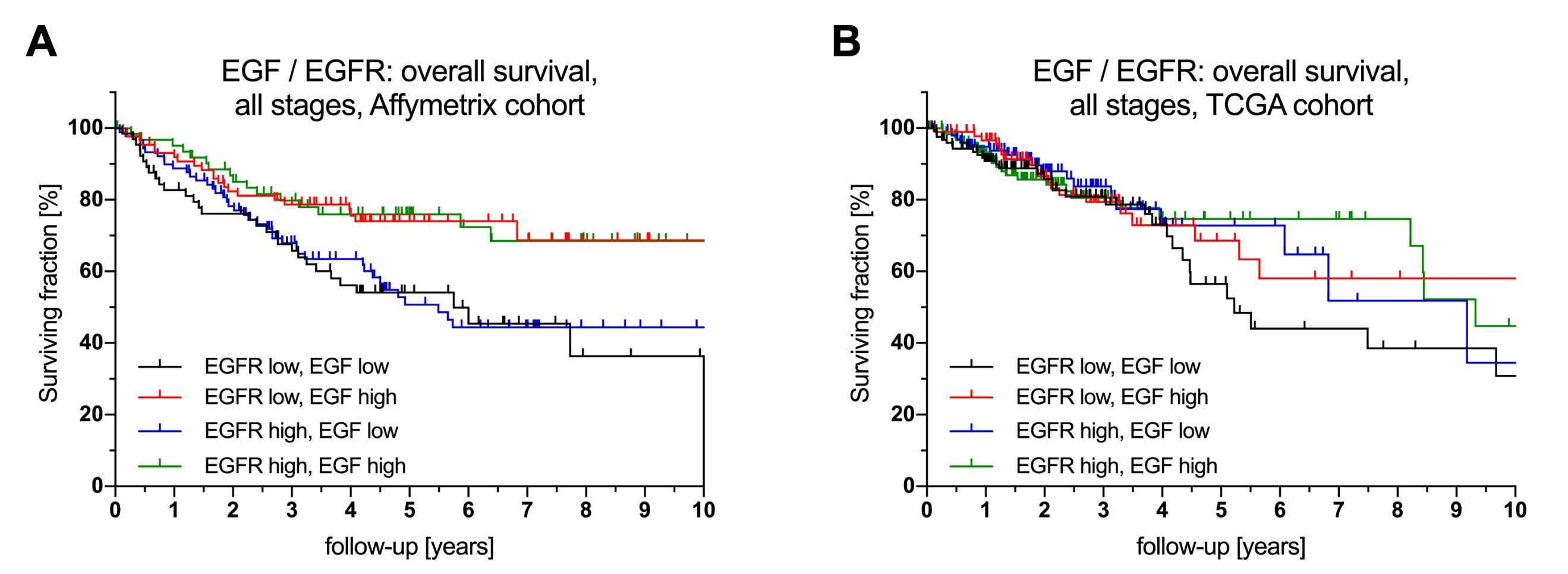


**Supplementary Figure S5: Kaplan Meier curves of serum EGF levels displaying the univariate survival analysis using the median as cutoff.**

Survival analysis for PlGF and EGF expression levels in disease of all stages in the Affymetrix cohort (A) and the TCGA cohort (B) for patients with high or low intratumoral EGFR and EGF expression (log-rank test). Abbreviations: EGF, epidermal growth factor; EGFR, epidermal growth factor receptor.


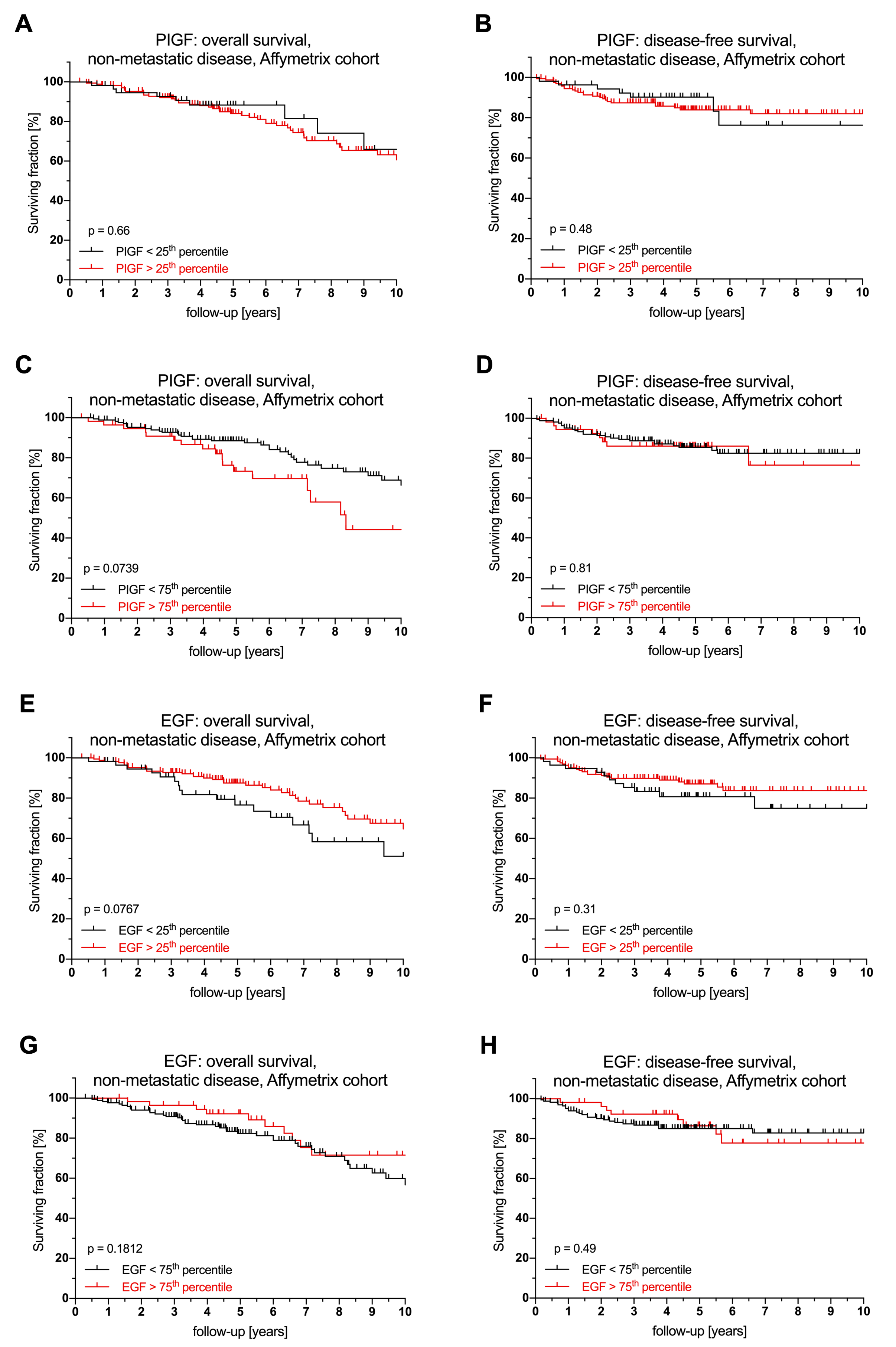


**Supplementary Figure S6. Prognostic effect of PlGF and EGF RNA expression on overall and disease-free survival in non-metastatic disease in the Affymetrix microarray cohort.**

Survival analysis for PlGF and EGF expression levels in non-metastatic disease in the Affymetrix cohort for overall survival and disease-free survival using the 25^th^ or 75^th^ percentile as cutoff (log-rank test). Abbreviations: PIGF, Phosphatidylinositol-glycan biosynthesis class F protein/Placental growth factor; EGF, epidermal growth factor.


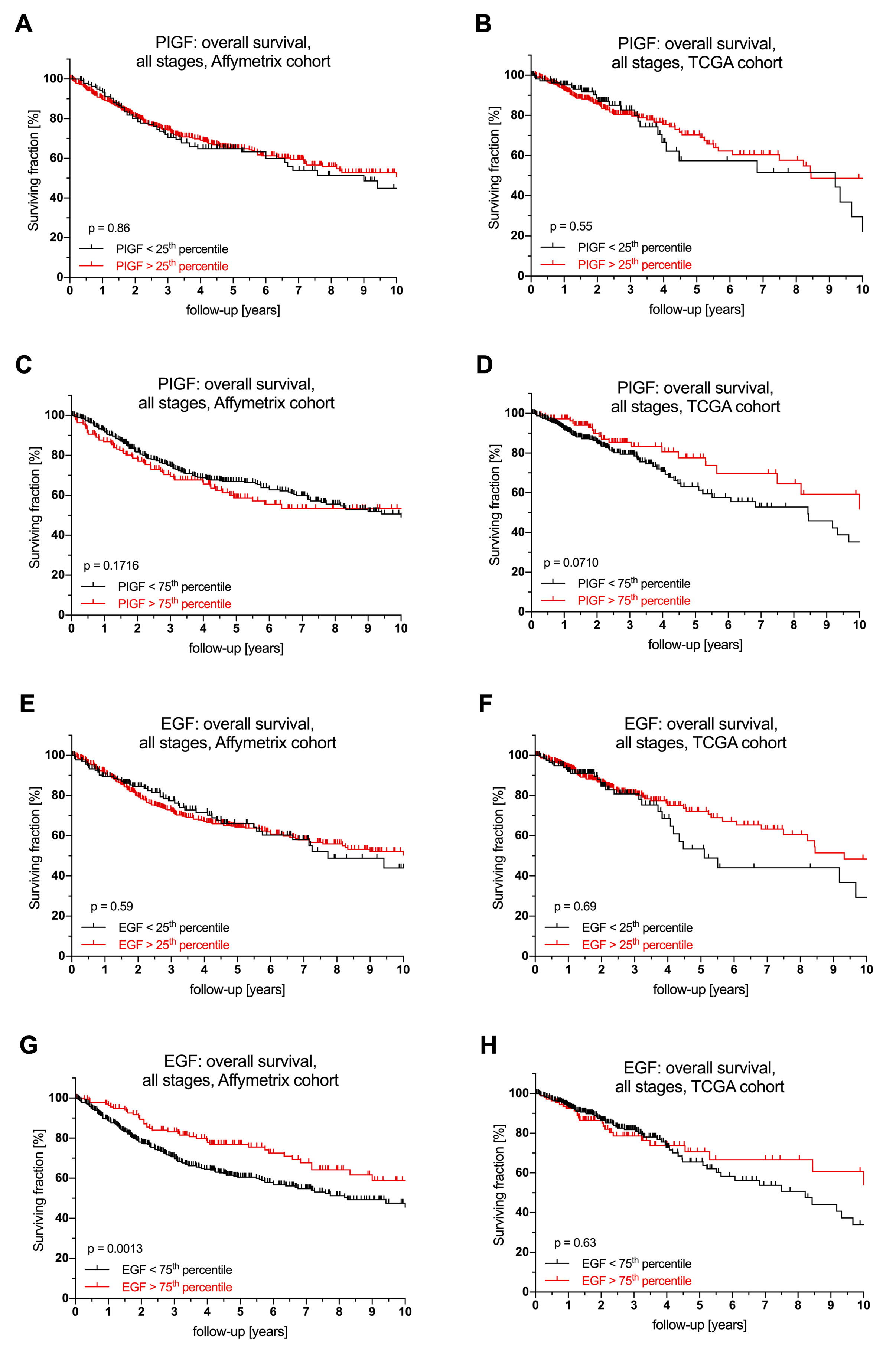


**Supplementary Figure S7. Prognostic effect of PlGF and EGF RNA expression on overall survival in patients of all stages in the Affymetrix microarray and TCGA RNAseq cohorts.**

Survival analysis for PlGF and EGF expression levels in patients of all stages in the Affymetrix and TCGA cohorts for overall survival using the 25^th^ or 75^th^ percentile as cutoff (log-rank test). Abbreviations: PIGF, Phosphatidylinositol-glycan biosynthesis class F protein/Placental growth factor; EGF, epidermal growth factor; TCGA, The Cancer Genome Atlas.
